# Supplementary material for: Cross-sectional study on intention to be vaccinated against Coronavirus Disease 2019 (COVID-19) in Benin and Senegal: A structural equation modeling (SEM)
Source: PLOS Glob Public Health. 2024 Mar 18;4(3):e0002868. doi: 10.1371/journal.pgph.0002868 (PMC10947661; doi:10.1371/journal.pgph.0002868)
Supplement: Attachment — Submitted filename: Reviewer report.docx [file pgph.0002868.s002.docx]

Title Cross-sectional study on Intention to be vaccinated against COVID-19 in Benin and Senegal: a structural equation modelling (SEM)

Reviewer: Solomon Abera

Reviewer's report:

This is an interesting manuscript with an important finding that could help to improve COVID-19 vaccination coverage be addressing the determinant factors. It also helps to support risk communication and health message development strategies and interventions to improve COVID-19 uptake in developing countries which is a cost-effective intervention. Additionally, the study will contribute to the scientific community to adopt the methods and apply to other context and vaccination program enhancement.

Saying this, the manuscript requires some grammatical editing to prepare for publications, reading through the manuscript, I have observed grammatical, scholarly tone, or additional unnecessary words. I hope that the author will review these to meet the level of scholarly writing requirement. Below please find my overall suggestions for considerations to strengthen the manuscript.

**Introduction**

- Line 57 and 60, uses different terminology to describe vaccine against COVID-19 vs vaccine against SARS-Cov2. Better to use the same terminology to be consistent. To be consistent with the title, it is appropriate to use vaccine against COVID-19.
- Line 83: word repetition. ‘’Vaccination against COVID-19 vaccination in Benin and Senegal’’

**Materials and methods**

Study type

- Line 94: data collection period for Senegal is from December 24, 2020, to January 16, 2021. However, elsewhere in the manuscript, it was mentioned that COVID-19 vaccination had been launched on March29, 2021? How could the data collection period be outside the period of the vaccination?
- Line 95: data collection period for Benin has been less than 3 months period, which is too short to get representative sample. It requires explanation of why this study period is specifically selected. Additionally, why different period for the two countries selected? It requires authors explanation why this study period selected?

**Sample size and sampling**

- Line 108: why P is set at 50%, the WHO target shows that 70% coverage by December 2020? Why was this proportion not used to calculate the sample size?
- Line 116: the quota sampling method was used to select the samples using age, gender, and religion as main variables; however, it would be good to explain why these variables selected. Reference has been added but the authors need to explain the reason here.

Line 95: Data collection period for Senegal started and completed before the country Launched COVID-19 vaccination. The data collection period is from December 24,2020 to January 16, 2021, however Senegal launched COVID-19 vaccine on March 29, 2021?

Line 96: Data collection period covers less than 3-month period for Benin, why is this very short period? How do you ensure representativeness of the participants? It is important to explain why this period is selected for the study? The study periods for the two countries are also different?

**Result**

Line335: the result indicated that women have slightly lower vaccination intention than men? Was this result statistically significant? Please show the statistical significance? for all comparisons, test of statistical significance needs to be done and added to the result, otherwise, it is not possible to tell readers whether the difference is statistically significant or note.
