# Supplementary material for: Cross-sectional study on intention to be vaccinated against Coronavirus Disease 2019 (COVID-19) in Benin and Senegal: A structural equation modeling (SEM)
Source: PLOS Glob Public Health. 2024 Mar 18;4(3):e0002868. doi: 10.1371/journal.pgph.0002868 (PMC10947661; doi:10.1371/journal.pgph.0002868)
Supplement: Attachment — Submitted filename: Response to Reviewers-1.docx [file pgph.0002868.s003.docx]

**Reviewer #1 : 1. In the questionnaire, was the respondent asked about his or her current vaccination status? People who have already been vaccinated (with at least one dose) will probably have a different viewpoint on many questions than those who "intend" to be vaccinated.**

There was no such question, as the study was conducted before the start of the vaccination campaign in Senegal (February 23, 2021). In Benin, the collection study began on the same day as the campaign (March 29, 2021).

**Reviewer #1 : 2. Line 115 – 116 under Methods, given how the quota sampling method was used, a multilevel SEM may be able to better address the correlation between, for example, respondents from the same region – because the survey could possibly reach many people within close communities by random dialing stratified by region.**

The quota approach was adopted in this study not to guarantee regional representativeness, but to ensure a balanced distribution of the sample across the different regions. The aim was to include a diversity of responses without necessarily aiming for statistical representativeness by region. So, while multilevel SEM can deal with the intra-class correlation typical of regionally stratified sampling, it may not be necessary in our context if we consider that correlation between participants in the same region is not a major bias factor for our analysis. Our main concern was to avoid any over- or under-representation of certain groups, and to ensure that our sample adequately covered the diversity of experiences and perspectives on a national scale.

*These elements have not been added to the Manuscript; instead, we have justified the choice of using the DWLS method* ***(Lines 332 -336).***

**Reviewer #1: 3. In the SEM analysis, a connection/effect was not observed between attitude and perception of vaccine efficacy in Benin because you didn't allow for such a causal path to exist in the proposed model structure? Similar for the causal path between attitude and behavioural control in Senegal, this path was not allowed in the proposed model structure. From my understanding of how the lavaan package works, if you did not specify the causal path, then it will assume the two items are independent by design?**

Our approach is slightly different. First, we used factor analysis. The AFE results, particularly the factor loadings (>0.7) [34], suggest the presence of associations between dimensions. This modelling process is the same as that described by Schumacker and Lomax. ***(Lines 323-327)***.

**Reviewer #1: 4. In discussion, while we don't see the model results give us a significant connection between perception of vaccine safety and vaccination intent in Senegal, how should we interpret this result and what are its implications? For instance, does this suggest that for the communication to be more effective, Senegal should focus less on communicating the safety/risk of vaccine because it is not a dominant factor, or is it because this result may be confounded with other factors such as personal attitude in the analysis? In general, more work needs to be done in communicating/interpreting the results and how the factors are connected to each other.**

This may suggest that vaccination communication campaigns should focus on other aspects instead. Alternatively, this result may indicate that vaccine safety concerns are already well managed by current information campaigns, and that these have succeeded in building a sufficient level of trust among most respondents.

*These elements have not been added to the Manuscript.*

**Reviewer #1: 5. In SEM, how did you handle the correlation between different questions in the survey? e.g., both attitude and behavioral control are significantly connected to social influence in Benin, but themselves are also significantly correlated.**

The correlations between the various dimensions were obtained using factor analysis. In fact, we applied a factorial analysis for each country, which enabled us to propose a different structure model for each country. These were then all materialized in the R implementation ***(Lines 323-327).***

**Reviewer #1: 6. In table 1, can you provide more details about why setting p (expected proportion) to 50%? What does this quantity mean in the context of this study and what are the implications by setting it to different values?**

The choice of P=50% is because sampling error is greatest when the proportion is 50%, giving the largest sample size needed for a given precision ***(Lines 113-115).***

**Reviewer #1: 7. Line 120, you mentioned about post-stratification statistical analyses were performed to adjust the data from the final sample. What exact post-stratification methods did you consider and implement?**

This is the margin calibration method ***(Line 142).***

**Reviewer #1: 8. Line 87, a 50.2% of female in the population is far from being described as "predominantly" - it's in fact quite an even split.**

This is indeed a balanced distribution. Correction made in the manuscript.

**Reviewer #1: 9. Line 345 – 346, are there any reasons for why more educated people are less intentional to get vaccinated? For example, did higher educated people also share lower perception of vaccine safety or more negative attitudes which led to a lower intention to vaccination?**

They perceive the associated risks differently, have less confidence in the authorities to manage the pandemic, and so on.

**Reviewer #1: 10. There is a gap of a couple months in the implementation between Senegal (Dec 2020) and Benin (March 2021). This should not be a huge concern but it’s worth mentioning any change or vaccine campaigns happening during this period that may have altered people’s perceptions.**

It's true that the two studies didn't start at the same time, but it's important to stress that in both countries the study started before the vaccination campaign began. Vaccination campaign start dates are mentioned in the introduction. ***(Line 68).***

**Reviewer #2: The Manuscript meets the PLOS Global public health's publication criteria and follows a scholarly writing outline. It is a well written manuscript with good level language and scholarly tone. The title, the objectives and methods are clearly described and follows a scientific writing fashion. Appropriated ethical clearness have secured and the paper has both research and publication ethics. Data is not available, but the author clearly mentioned that the data will be available up on request.**

All data and analysis codes are now freely available from this link: <https://doi.org/10.6084/m9.figshare.24574432.v2> ***(Line 677).***

**Reviewer #3: This is a well-written manuscript. As I am not a native English speaker, my assessment of the quality of the English used in this manuscript may be inadequate. The methodology is described in detail and the conclusions are realistic.
The authors have not made public all the data required to reproduce the results of their study. It would be good if they could explain why.
Thank you.**

All data and analysis codes are now freely available from this link: <https://doi.org/10.6084/m9.figshare.24574432.v2> ***(Line 677).***

**Reviewer #4: Coronavirus disease-19 is a pathology which has evoked important discussions in health from local to international levels and it's not ending anytime soon. I am optimistic this publication will improve the advocacy and management of COVID-19 and future pandemics not just in the global south but also the global north. I commend the authors for putting this together, it is excellent research and well written from authors that come from countries whose primary language is not English.**

Nothing to report.

**Rewiewer #5: 1. Line 94: data collection period for Senegal is from December 24, 2020, to January 16, 2021. However, elsewhere in the manuscript, it was mentioned that COVID-19 vaccination had been launched on March29, 2021? How could the data collection period be outside the period of the vaccination?**

Data collection effectively preceded the start of vaccination campaigns in both countries. But as of December 2020, COVID-19 vaccines were already available, so the population was able to declare its intention to vaccinate from that point onwards ***(Line 34 and line 68).***

**Rewiewer #5: 2. Line 95: data collection period for Benin has been less than 3 months period, which is too short to get representative sample. It requires explanation of why this study period is specifically selected. Additionally, why different period for the two countries selected? It requires authors explanation why this study period selected?**

The representativeness of the sample in the Benin study is not influenced by the duration of data collection. A sample can be considered representative if sampling procedures are correctly designed and applied, regardless of the duration of data collection.

Differences in data collection periods between Benin and Senegal are due to logistical constraints, notably delays in obtaining ethical authorizations, which are not always synchronized between countries.

*These explanations have not been added to the Manuscript.*

**Rewiewer #5: 3. Line 108: why P is set at 50%, the WHO target shows that 70% coverage by December 2020? Why was this proportion not used to calculate the sample size?**

The choice of P=50% is because sampling error is greatest when the proportion is 50%, giving the largest sample size needed for a given precision ***(Lines 113 – 115).***

**Rewiewer #5: 4. Line 116: the quota sampling method was used to select the samples using age, gender, and religion as main variables; however, it would be good to explain why these variables selected. Reference has been added but the authors need to explain the reason here.**

The selection of these quota variables considers the significant regional disparities in health practices, access to health services and attitudes towards vaccination that can influence individual decisions. In addition, age is a critical factor, since perceptions and behaviors towards health risks often differ between younger and older individuals, thus affecting intention to vaccinate. Finally, gender is included as a key variable because of the marked differences in social norms and roles that shape perceptions of vaccination and health decision-making. The use of these specific variables ensures a balanced and nuanced representation of the study population, which is essential for the accuracy and relevance of our analyses concerning vaccination intention ***(Lines 122- 130)***.

**Rewiewer #5: 5. Line335: the result indicated that women have slightly lower vaccination intention than men? Was this result statistically significant? Please show the statistical significance? for all comparisons, test of statistical significance needs to be done and added to the result, otherwise, it is not possible to tell readers whether the difference is statistically significant or note.**

Differences are statistically significant ***(lines 360 – 362)****.*

We hope that our responses and changes to the manuscript are satisfactory and meet the expectations of the review committee. We welcome further discussion and are prepared to make further changes if necessary.

We appreciate the attention given to our work and look forward to our study making a significant contribution to the existing literature.

Best regards,

Ibrahima GAYE
